# Remifentanil attenuates endoplasmic reticulum stress and inflammatory injury in LPS-induced damage in HK-2 cells

**DOI:** 10.1080/0886022X.2022.2134028

**Published:** 2022-10-19

**Authors:** Yixiu Yan, Na Zhu, Dan Jin, Feihong Lin, Ya Lv

**Affiliations:** Department of Anesthesiology, The First Affiliated Hospital of Wenzhou Medical University, Wenzhou, Zhejiang, P. R. China

**Keywords:** Remifentanil, endoplasmic reticulum stress, renal injury, sepsis, inflammation

## Abstract

Renal injury is a fatal complication in critically ill patients with sepsis. As an ultrashort-acting synthetic opioid derivative, remifentanil has been reported to mitigate renal injury and sepsis. Nevertheless, whether remifentanil also suppresses sepsis-triggered renal injury is uncertain. The aim of this study was to investigate the effect of remifentanil on endoplasmic reticulum stress (ERS) and inflammatory response in an *in vitro* lipopolysaccharide (LPS)-stimulated renal tubular epithelial cell (HK-2) model and its mechanism. The viability of HK-2 cells with the absence or presence of LPS treatment was surveyed by cell counting kit-8 assay. Under the condition of LPS treatment, apoptosis was appraised by TUNEL assay and western blot. Levels of inflammatory factors were estimated though corresponding kits. Western blot tested the expression of toll-like receptor 4 (TLR4)/nuclear factor-kappaB (NF-κB) signaling-associated proteins. Also, the expression of ERS-related proteins was detected by western blot. Further, ERS inducer tunicamycin (TM) was added and the aforementioned experiments were conducted again. The results underlined the protective effects of remifentanil on LPS-evoked viability injury, inflammation, activation of TLR4/NF-κB signaling and ERS in HK-2 cells. Moreover, the impacts of remifentanil on the biological events of LPS-insulted HK-2 cells were all reversed by TM administration. To conclude, remifentanil might have a remarkable ameliorative effect on sepsis-induced renal injury, which implied the potential of remifentanil-based drug therapy in sepsis-induced renal injury.

## Introduction

Sepsis is the most predominant cause of death in patients admitted to the intensive care unit [1]. In addition, sepsis can cause excessive systemic inflammatory response through uncontrolled release of inflammatory mediators due to infection [2,3]. In addition to the increased inflammatory response and immunosuppression, the destabilization of blood flow, multiple tissue damage, and multi-organ dysfunction also occur in the process of sepsis due to cytokine dysregulation [4–6]. One of the organs most vulnerable to sepsis is the kidney, and acute kidney injury (AKI) is the most frequent and severe complication of sepsis [7]. Sepsis-induced AKI accounts for about 50% of the incidence of AKI caused by all causes, which is characterized by high morbidity and mortality. It is reported that the fatality rate is as high as 70%, and it also increases the risk of developing chronic kidney disease and end-stage renal disease [8–11]. The pathogenesis of AKI has not been fully elucidated. Inflammatory reaction, oxidative stress reaction, microvascular endothelial dysfunction, renal tubule epithelial cell injury, and other potential mechanisms of AKI [12]. Current therapeutic modalities are primarily dependent on renal-replacement therapy [13]. Also, drugs including agomelatine, ticagrelor, and dexmedetomidine are emerged in the treatment of sepsis-induced renal injury [14–16]. Hence, figuring out the effective drug therapy for sepsis-evoked renal injury is capable of declining sepsis-associated death rate. Of particular concern is that the major cause of AKI in sepsis is lipopolysaccharide (LPS)-mediated apoptosis of renal tubular epithelial cells [17,18]. Therefore, the use of LPS-mediated renal tubular epithelial cells as an *in vitro* model of sepsis-induced kidney injury is of research significance.

Remifentanil is an ultra-short-acting synthetic opioid derivative of piperidine [19,20]. It can be metabolized by nonspecific plasma and tissue esterase and is thus independent of hepatic and/or renal function [21,22]. It has been reported that remifentanil inhibited LPS-induced inflammatory response in human aortic endothelial cells via PARP-1/NF-κB pathway [22]. Remifentanil also protected against LPS-induced oxidative damage in cardiomyocytes through partially downregulating PKCβ2 activation and inhibiting autophagy [23] and attenuated LPS-induced acute lung injury via downregulating NF-κB pathway [24]. Of note, remifentanil has been intensively applied in therapy for sepsis [22,25,26]. The apoptosis was decreased by regulating the expression of apoptosis and endoplasmic reticulum stress (ERS)-related proteins in LPS-induced AKI [27]. C/EBP homologous protein (CHOP) is an important mediator of ERS-induced cell and organ injury and LPS-induced AKI is associated with ER stress and elevated CHOP [28]. Remifentanil alleviated the intestinal I/R injury by its PDIA3-mediated antioxidant and anti-ER stress properties [29]. Remifentanil protected against ischemia/reperfusion injury in heart by inhibiting ERS [30]. Nevertheless, the potential effects and mechanisms of remifentanil in sepsis-induced kidney injury have not been fully investigated.

Sepsis is prone to AKI, and the mechanism is related to the activation of toll-like receptor 4 (TLR4)/nuclear factor-kappaB (NF-κB) signaling pathway by bacterial products to produce a large number of inflammatory factors [31–34]. TLR4 is a pattern recognition receptor that acts as a sensor of LPS induced inflammation, activating inflammatory factors [35–37] to further activate the NF-κB pathway, which leads to nucleation of p65 egg white and increase of the pro-inflammatory factor tumor necrosis factor alpha (TNF-α), interleukin 1-beta (IL-1β), and interleukin 6 (IL-6) expression [38–42]. TLR4/NF-κB signaling pathway is one of the most important mechanisms leading to sepsis associated AKI [43]. A study showed that remifentanil protected against myocardial ischemia/reperfusion (I/R) injury by miR-206-3p/TLR4/NF-κB signaling pathway [44]. We speculated that remifentanil might alleviated the sepsis-induced kidney injury through TLR4/NF-κB signaling pathway.

Consequently, the aim of this study was to investigate the effects of remifentanil on LPS-induced ERS and inflammatory response in renal epithelial cells and its mechanisms. It was hypothesized that remifentanil might mitigate LPS-insulted HK-2 cell injury through blocking ERS and inflammation. The present study was the first to reveal the impacts of remifentanil on sepsis-induced renal injury and clarify the mechanism in which remifentanil regulated sepsis-induced renal injury through mediating ERS.

## Materials and methods

### Cell culture and treatment

Human renal tubular epithelial cells (HK-2) were obtained from BeNa Culture Collection. Cells were grown in RPMI-1640 medium (Beijing Solarbio Science & Technology Co., Ltd.) containing 10% FBS at 37 °C with 5% CO_2_. In addition, cells were treated by different doses of remifentanil (0.625, 1.25, and 2.5 μM; Yichang Humanwell Pharmaceutical Co., Ltd.) for 24 h as needed [22,23]. Tunicamycin (TM; 0.5, 1, 5, and 10 μg/ml; Abcam) [45–48] was used as an ERS inducer to pretreat cells at 37 °C for 2 h before treatment with remifentanil.

### Cell counting kit-8 assay

HK-2 cells (5 × 10^3^/well) were placed in 96-well plates. Upon cell apposition, cells were processed according to grouping. Afterwards, 10 μl cell counting kit-8 (CCK-8) reagent (Beijing Solarbio Science & Technology Co., Ltd.) was added to each well and incubated for 4 h. Finally, the absorbance at 450 nm was measured by enzyme marker (Thermo Fisher Scientific, Inc.).

### TUNEL staining

HK-2 cells were stained for apoptosis with the one-step TUNEL apoptosis assay kit (Shanghai Beyotime Biotechnology Co., Ltd.) according to the manufacturer’s instructions. Cells were fixed with 3.7% paraformaldehyde and incubated with 0.5% Triton X-100 at 4 °C for 5 min. Subsequently, cells were incubated with TUNEL solution at room temperature protected from light for 1 h. Finally, nuclei were labeled with DAPI and cells were photographed by fluorescence microscopy (magnification ×200; EUROIMMUN, Lubeck, Germany).

### Western blotting

HK-2 cells were lysed with RIPA buffer (Shanghai Beyotime Biotechnology Co., Ltd.) containing protease inhibitors to obtain total protein. After separation with 10% sodium dodecyl sulfate-polyacrylamide gels, protein samples were transferred to polyvinylidene difluoride membranes. After blocking for 2 h at room temperature, the membranes were incubated with primary antibodies at 4 °C overnight. Subsequently, the corresponding secondary antibodies (ab6734, 1/1000, Abcam) were added and incubated for another 1 h. Protein signals were visualized using an ECL kit (Thermo Fisher Scientific, Inc.) and quantified using Image-Pro Plus version 6.0 software (Roper Technologies, Inc.). Primary antibodies used in this experiment were against B cell lymphoma-2 (Bcl-2; ab32124, 1/1000, Abcam), BCL-2 associated X (Bax; ab32503, 1/1000, Abcam), cleaved-Poly (ADP-ribose) polymerase (c-PARP; ab32064, 1/1000, Abcam), PARP (ab191217, 1/1000, Abcam), C-caspase3 (ab32042, 1/500, Abcam), caspase3 (ab32351, 1/5000, Abcam), TLR4 (ab13556, 1/1000, Abcam), myeloid differentiation primary response 88 (MyD88; ab133739, 1/1000, Abcam), TNF receptor-associated factor 6 (TRAF6; ab33915, 1/2000, Abcam), phosphorylated-p65 (p-p65; ab76302, 1/1000, Abcam), p65 (ab32536, 1/1000, Abcam), NOD-like receptor family pyrin domain containing 3 (NLRP3; ab263899, 1/1000, Abcam), phosphorylated protein kinase RNA-activated-like endoplasmic reticulum (ER) kinase (p-PERK; #3179, 1/1000, Cell Signaling Technology), PERK (ab229912, 1/1000, Abcam), phosphorylated alpha-subunit of eukaryotic translation initiation factor 2 (p-eIF2α; ab32157, 1/1000, Abcam), eIF2α (ab169528, 1/1000, Abcam), Activating transcription factor 6 (ATF6; ab227830, 1/1000, Abcam), C/EBP homologous protein (CHOP; #5554, 1/1000, Cell Signaling Technology), and GAPDH (ab9485, 1/2500, Abcam).

### Inflammatory factor levels

The expression levels of TNF-α, IL-6, and IL-1β in the HK-2 cell supernatant were measured according to the instructions by sequentially using the human TNF-α ELISA kit (#RAB1089; Sigma, Merck), human IL-6 ELISA kit (#RAB0307; Sigma, Merck), and human IL-1β ELISA kit (#RAB0273; Sigma, Merck).

### Statistical analysis

Statistical analysis was performed with the assistance of GraphPad Prism 5.0 (GraphPad software, Inc.). In the bar charts, comparisons of differences between multiple groups were calculated using one-way ANOVA with Tukey’s test. The experiments for each group were repeated at least three times. Differences were considered statistically significant when expressed as *p < 0.05, **p < 0.01, and ***p < 0.001.

## Results

### Remifentanil inhibits LPS-induced apoptosis in HK-2 cells

**Figure 1.**
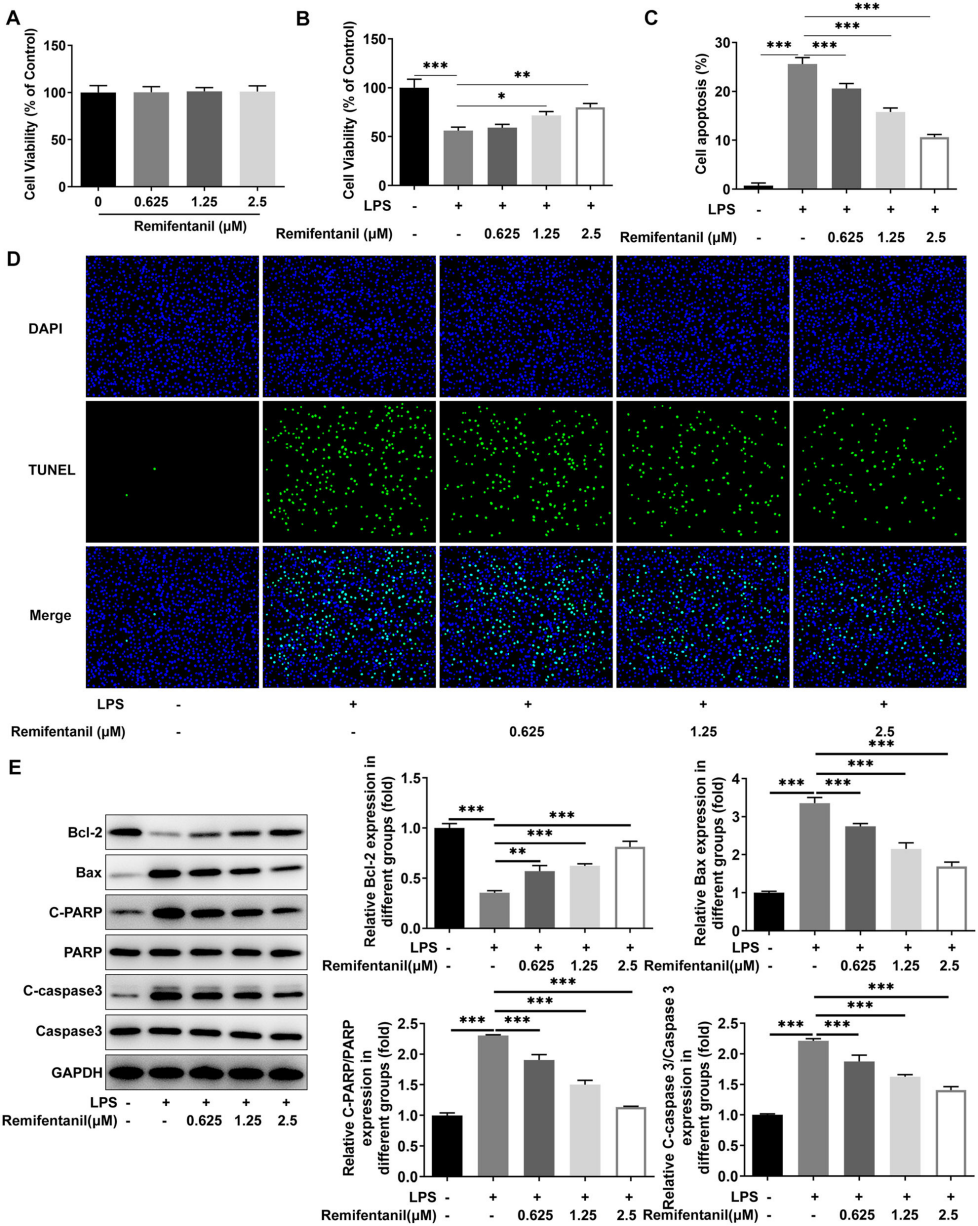
Remifentanil inhibits LPS-induced apoptosis in HK-2 cells. (A) CCK-8 clarified the effects of different concentrations of remifentanil on HK-2 cell viability. (B) CCK-8 clarified the effects of different concentrations of remifentanil on cell viability in LPS-induced HK-2 cells. (C and D) Apoptosis was detected by using TUNEL staining. (E) Expression of apoptosis-related proteins (Bcl-2, Bax, PARP, and caspase3) was detected by western blot. **p < 0.01 and ***p < 0.001. *n* = 3. Bcl-2: B cell lymphoma-2; Bax: BCL-2 associated X; c-PARP: cleaved-poly(ADP-ribose) polymerase; PARP: poly(ADP-ribose) polymerase.

First, the viability of HK-2 cells was detected following administration of different concentrations of remifentanil (0.625, 1.25, and 2.5 μM). The results showed that no apparent effects on HK-2 cell viability were noticed exposed to ascending doses of remifentanil (Figure 1(A)); however, enhancive doses of remifentanil concentration-dependently exacerbated the impaired viability of HK-2 cells upon exposure to LPS (Figure 1(B)). Cell apoptosis was detected by TUNEL staining and western blot. The experimental data manifested that LPS induction prominently strengthened the apoptosis rate, but apoptosis was markedly attenuated by different concentrations of remifentanil treatment, of which 2.5 μM remifentanil had the best inhibitory effect (Figure 1(C,D)). Also, it was found that due to LPS induction, anti-apoptotic protein Bcl-2 expression was distinctly reduced, while pro-apoptotic protein Bax expression and c-PARP/PARP and c-caspase3/caspase3 expression was prominently aggrandized. Notably, Bcl-2, Bax, c-PARP/PARP, and c-caspase3/caspase3 expression was all dose-dependently reversed by remifentanil administration (Figure 1(E)). To sum up, remifentanil eased LPS-evoked viability injury and apoptosis in HK-2 cells.

### Remifentanil reduces LPS-induced inflammation and ERS level in HK-2 cells

**Figure 2.**
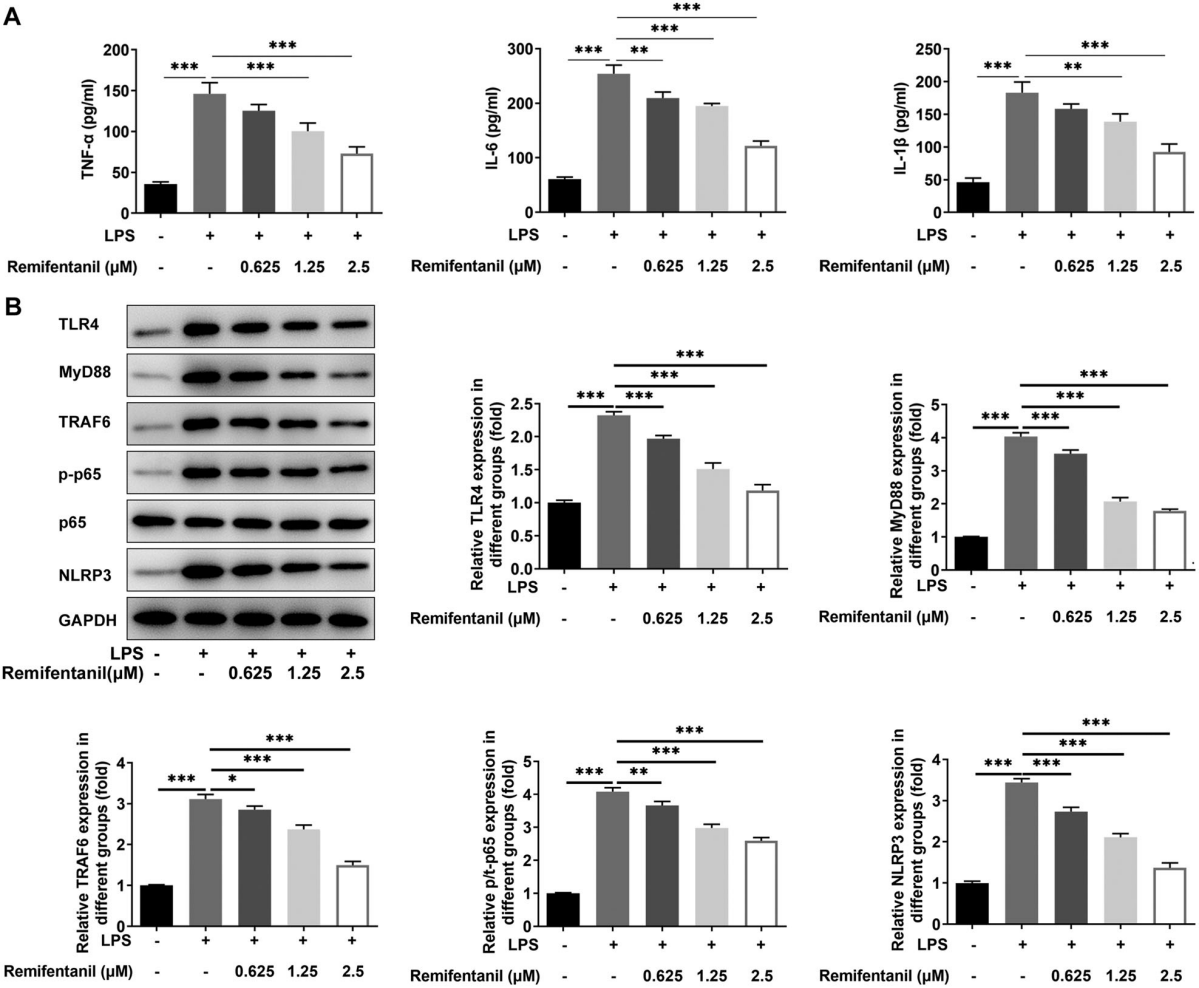
Remifentanil reduces LPS-induced inflammation in HK-2 cells. (A) Inflammatory factor (TNF-α, IL-6, and IL-1β) levels were examined by reagent kits. (B) The expression of TLR4/NF-κB signaling-related proteins was detected by western blot, including TLR4, MyD88, TRAF6, p/t-p65, and NLRP3. *p < 0.05, **p < 0.01, and ***p < 0.001. *n* = 3. TNF-α: tumor necrosis factor-alpha; IL-6: interleukin 6; IL-1β: interleukin-1beta; TLR4: toll-like receptor 4; MyD88: myeloid differentiation primary response 88; TRAF6: tumor necrosis factor (TNF) receptor-associated factor 6; P-p65: phosphorylated-p65; NLRP3: NOD-like receptor family pyrin domain containing 3.

Subsequently, levels of inflammatory factors and inflammation-related proteins were measured. The levels of TNF-α, IL-6, and IL-1β were notably augmented in the LPS-induced group by contrast with the control group. However, TNF-α, IL-6, and IL-1β levels were lessened with the administration of increasing concentrations of remifentanil relative to the LPS group (Figure 2(A)). Similarly, TLR4, MyD88, TRAF6, NLRP3, and p/t-p65 protein expression was fortified after LPS induction and suppressed after remifentanil treatment (Figure 2(B)).

**Figure 3.**
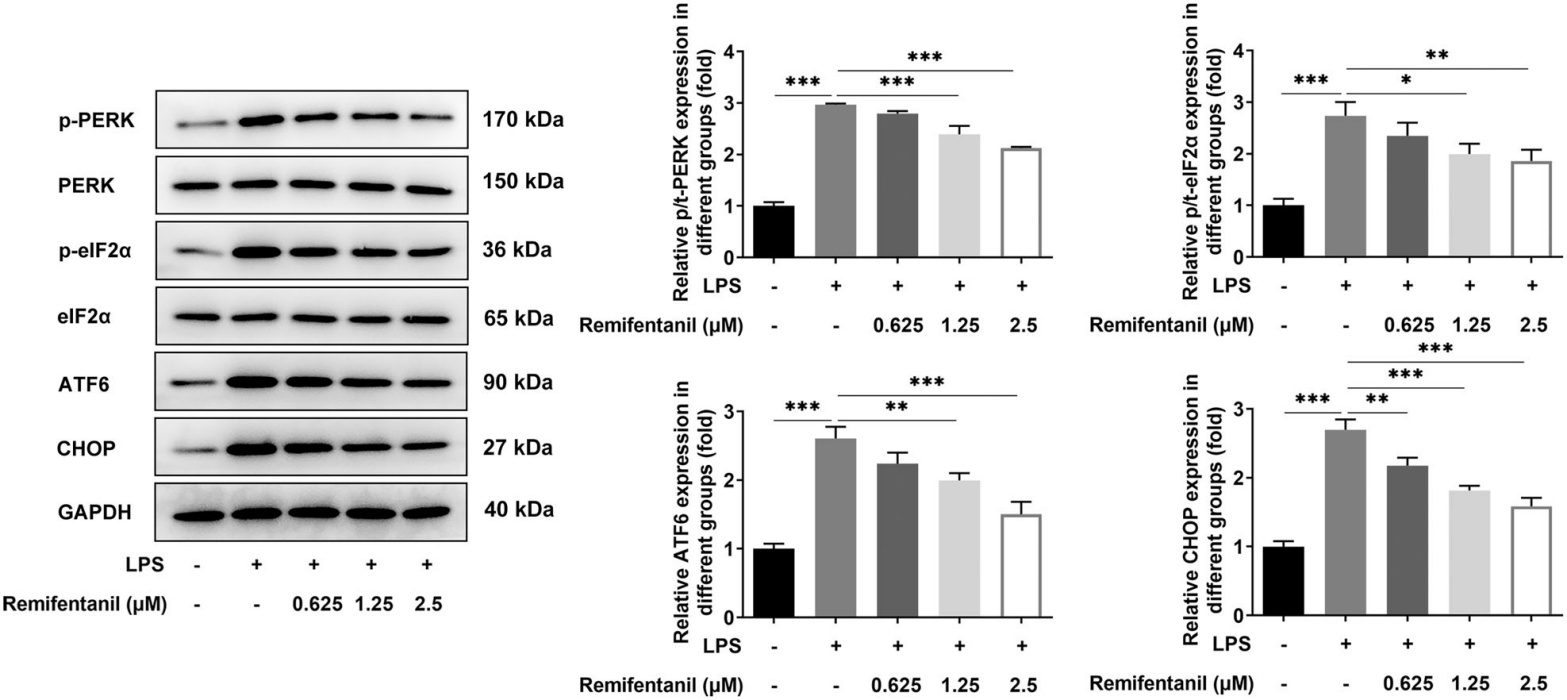
Remifentanil attenuates LPS-induced ERS level in HK-2 cells. Expression of ERS-related proteins was detected by western blot, including p/t-PERK, p/t-eIF2α, ATF6, and CHOP. *p < 0.05, **p < 0.01, and ***p < 0.001. *n* = 3. P-PERK: phosphorylated protein kinase RNA-activated-like endoplasmic reticulum (ER) kinase; PERK: protein kinase RNA-activated-like endoplasmic reticulum (ER) kinase; P-eIF2α: phosphorylated alpha-subunit of eukaryotic translation initiation factor 2; eIF2α: alpha-subunit of eukaryotic translation initiation factor 2; ATF6: activating transcription factor 6; CHOP, C/EBP homologous protein.

ERS level was estimated by western blot analysis of the expression of ERS-associated proteins. LPS induction increased the expression of p/t-PERK, p/t-eIF2α, ATF6, and CHOP by contrast with the control group. However, remifentanil protection inhibited the expression of p/t-PERK, p/t-eIF2α, ATF6, and CHOP in a dose-dependent manner relative to the LPS group (Figure 3). Anyway, remifentanil alleviated inflammation and ERS in HK-2 cells under LPS stimulation.

### Remifentanil inhibits LPS-induced apoptosis and inflammation levels in HK-2 cells by suppressing ERS

**Figure 4.**
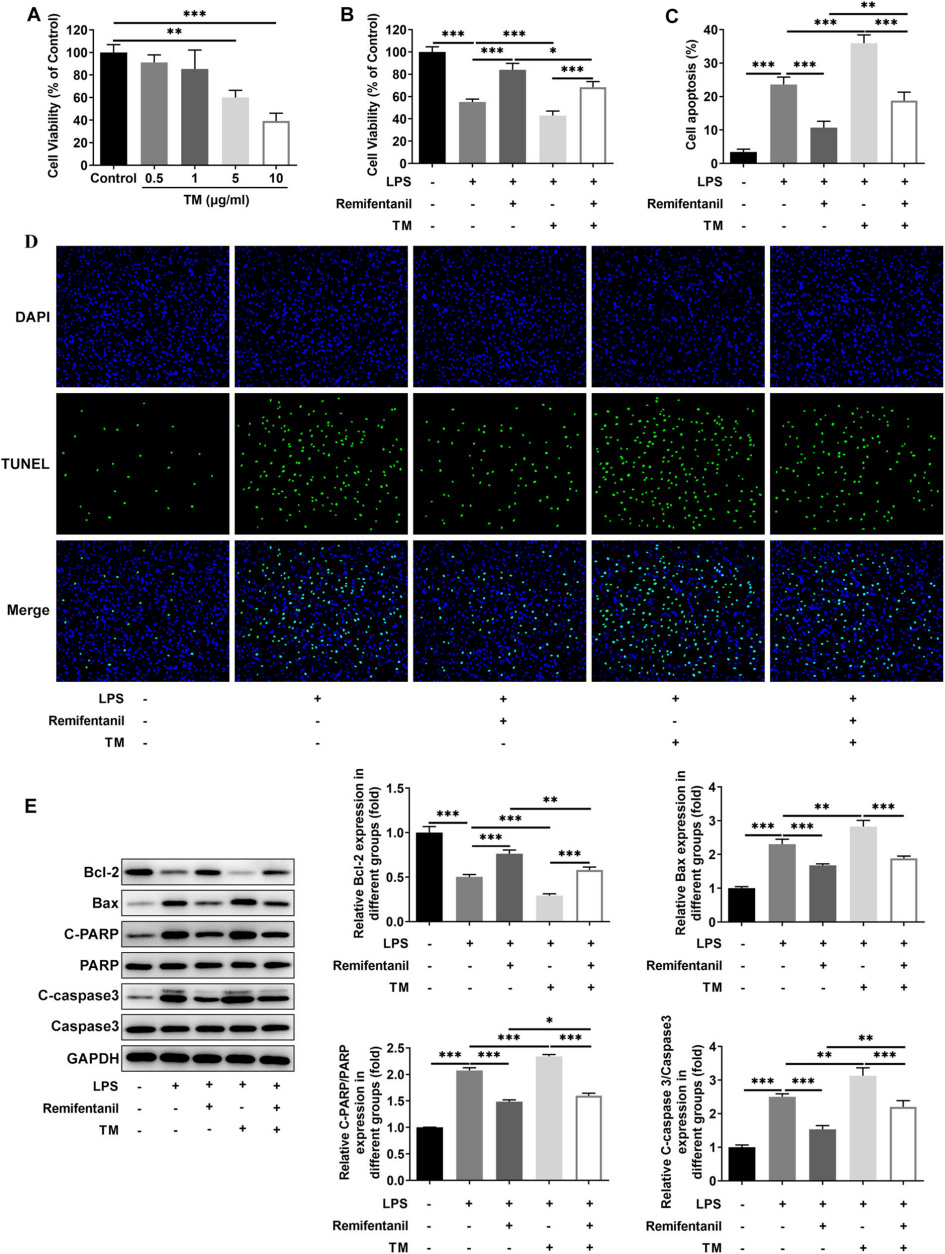
Remifentanil inhibits LPS-induced apoptosis in HK-2 cells by suppressing ERS. (A and B) CCK-8 examined HK-2 cell viability. (C and D) Apoptosis was detected by using TUNEL staining. (E) Expression of apoptosis-related proteins (Bcl-2, Bax, PARP, and caspase3) was detected by Western blot. *p < 0.05, **p < 0.01, and ***p < 0.001. *n* = 3. TM: Tunicamycin; Bcl-2: B cell lymphoma-2; Bax: BCL-2 associated X; c-PARP: cleaved-poly(ADP-ribose) polymerase; PARP: poly(ADP-ribose) polymerase.

**Figure 5.**
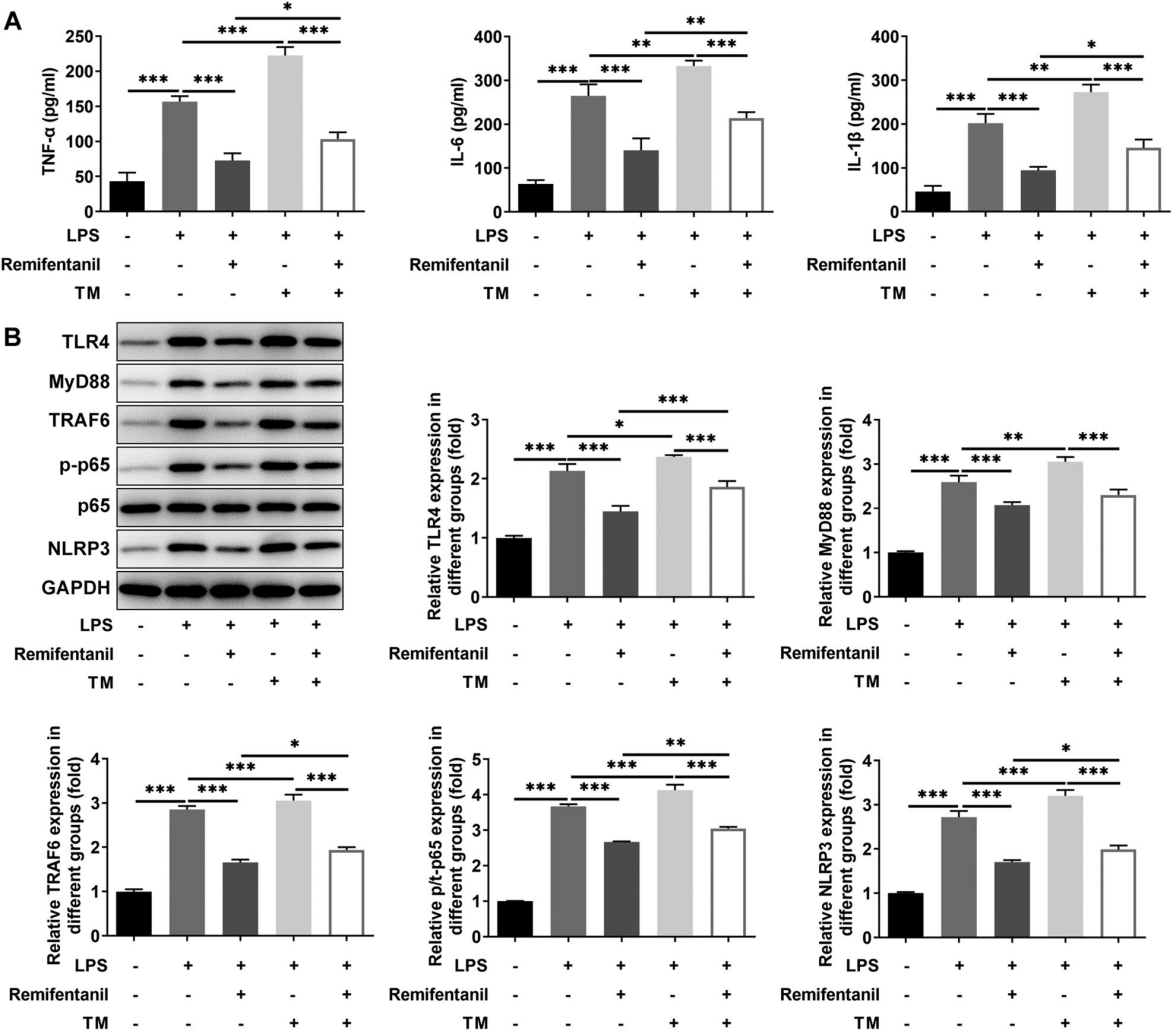
Remifentanil inhibits LPS-induced inflammation in HK-2 cells by suppressing ERS. (A) Inflammatory factor (TNF-α, IL-6, and IL-1β) levels were detected by reagent kits. (B) The expression of TLR4/NF-κB signaling-related proteins were detected by western blot, including TLR4, MyD88, TRAF6, p/t-p65, and NLRP3. *p < 0.05, **p < 0.01, and ***p < 0.001. *n* = 3. TM: tunicamycin; TNF-α: tumor necrosis factor-alpha; IL-6: interleukin 6; IL-1β: interleukin-1beta; TLR4: toll-like receptor 4; MyD88: myeloid differentiation primary response 88; TRAF6: tumor necrosis factor (TNF) receptor-associated factor 6; p-p65: phosphorylated-p65; NLRP3: NOD-like receptor family pyrin domain containing 3.

The viability of HK-2 cells following administration of different concentrations of ERS inducer TM was detected, which showed that no apparent effects on HK-2 cell viability were observed at 0.5 and 1 μg/ml TM and HK-2 cell viability was decreased by TM at 5 and 10 μg/ml. So, 1 μg/ml TM was chosen for subsequent experiment (Figure 4(A)). To examine whether remifentanil functioned in LPS-elicited HK-2 cell injury via modulating ERS, 1 μg/ml TM, and 2.5 μM remifentanil were chosen for subsequent experiments. Remifentanil had a protective effect on LPS-induced HK-2 cell viability injury by contrast with the LPS group, but the addition of TM noticeably suppressed cell viability (Figure 4(B)). In addition, the inhibitory effect of remifentanil on LPS-induced HK-2 cell apoptosis was markedly reversed by the presence of TM (Figure 4(C,D)). Similarly, the presence of TM significantly reversed the fortified Bcl-2 expression and the lessened Bax, c-PARP/PARP, and c-caspase3/caspase3 expression on account of remifentanil administration in HK-2 cells under LPS stimulation (Figure 4(E)). Further, TNF-α, IL-6, and IL-1β levels were dramatically increased in the LPS + Remifentanil + TM group in comparison to the LPS + Remifentanil group (Figure 5(A)). Meanwhile, the inhibitory effect of remifentanil on TLR4/NF-κB inflammatory pathway-related proteins (TLR4, MyD88, TRAF6, NLRP3, and p/t-p65) expression in LPS-injured cells was effectively reversed by TM (Figure 5(B)). TM suppressed the viability, while promoted apoptosis and inflammation of LPS-induced HK-2 cells. Overall, remifentanil ameliorated ERS to reduce apoptosis and inflammation in LPS-insulted HK-2 cells.

## Discussion

The present study aimed to provide evidence on whether remifentanil could attenuate sepsis-induced renal tubular epithelial cell injury and suggest a possible mechanism for it. The study used a recognized sepsis-induced renal tubular epithelial cell model to evaluate the effects of remifentanil on sepsis-triggered renal injury. The effects of remifentanil on LPS-induced apoptosis, inflammation, and ERS were investigated, while the associated mechanism was deeply investigated by the addition of the ERS inducer TM.

AKI, a common complication of sepsis, has a mortality rate of up to 40% [49]. In recent years, more researchers have considered that the main causative factors of sepsis-induced kidney injury are renal cell apoptosis and inflammation [50–52]. Hence, inhibition of cell apoptosis and inflammation becomes the major focus of treatment for sepsis-induced kidney injury. LPS is highly immunostimulatory in mammals which can trigger septic symptoms on its own [53]. In our study, LPS was shown to remarkably induce apoptosis in renal epithelial cells, cut down Bcl-2 expression, and augment Bax, C-caspase3/caspase3 expression. PARP is one of the pathogenic factors involved in septic cell dysfunction, inflammation, and organ failure [54]. In this study, C-PARP/PARP expression was also noted to be distinctly enhanced after LPS induction.

Furthermore, TLR4 works as an LPS sensor, and its activation of inflammatory factors in turn causes renal injury; it also activates the NF-κB pathway associated with the production of pro-inflammatory cytokines TNF-α, IL-6, and IL-1β [32]. In the present study, the levels of inflammatory factors TNF-α, IL-6, and IL-1β were dramatically elevated by LPS induction. At the same time, the protein levels of TLR4, p/t-p65, and NLRP3 were notably increased. The changes in these indicators were consistent with those in sepsis-induced renal tubular epithelial cell injury [55].

Remifentanil is an opioid receptor agonist with anti-inflammatory and anti-sympathetic effects to improve microcirculation [22,56]. It is well documented that remifentanil is intensively applied in therapy for sepsis for its anti-inflammatory property [22–24]. In the present study, remifentanil exerted no influence on the viability of untreated renal tubular epithelial cells. Moreover, LPS-damaged cell viability was reversed by remifentanil administration. At the same time, remifentanil had a marked ameliorative effect of LPS-evoked apoptosis, inflammatory response in HK-2 cells in a dose-dependent manner. Further, the impacts of LPS stimulation on apoptosis-related factors were also counteracted after the supplementation of remifentanil. These effects results were consistent with the trend shown by remifentanil in LPS-induced oxidative damage of cardiomyocytes and sepsis-associated inflammation study [22,23].

Notably, ER, as a subcellular organelle, is involved in several physiological activities. Under stress and inflammatory conditions, the ER may lose its functional homeostasis and trigger cell damage or even apoptosis once the stress is overwhelmed [57]. Meanwhile, substantial evidence has suggested that ERS is involved in the pathogenesis of sepsis [58,59]. Thus, in our study, ERS-related proteins were investigated. Intriguingly, recent literatures have corroborated that remifentanil is capable of relieving ERS in cardiac ischemia/reperfusion injury [30,60,61] and intestinal ischemia/reperfusion [29]. In the same way, the present findings also revealed that the expression of p/t-PERK, p/t-eIF2α, ATF6, and CHOP was remarkably increased after LPS induction when compared with the control group. However, with the persistent increase in remifentanil dose, the expression of the above proteins was suppressed compared with the LPS group. This finding indicated that remifentanil could ease LPS-induced ERS in renal tubular epithelial cells.

More importantly, the suppressive effects of remifentanil on apoptosis and expression of apoptosis-related factors in LPS-injured renal tubular epithelial cells were reversed by the presence of ERS inducer TM. As expected, the effects of remifentanil on inflammation-related indicators including TNF-α, IL-6, IL-1β, TLR4, p/t-p65, and NLRP3 were all reversed by TM stimulation. Taken together, remifentanil could attenuate ERS and thus reducing LPS-induced inflammatory injury in renal tubular epithelial cells.

## Conclusion

Remifentanil effectively ameliorated excessive apoptosis and inflammatory response during LPS-induced renal tubular epithelial cell injury through modulation of ERS. Our findings were the first to uncover the role of remifentanil and elaborate the association between remifentanil and ERS in LPS-induced renal tubular epithelial cells, which might provide novel insights into the application of remifentanil in human diseases, contribute to a deeper understanding of the mechanism underlying remifentanil as well as provide a theoretical basis for drug therapy dependent on remifentanil in septic kidney injury diseases. However, the *in vitro* experiments in this paper are insufficient, and the experiments in animals will be conducted in future.
